# Correlates of oral pre-exposure prophylaxis cessation among men who have sex with men in China: implications from a nationally quantitative and qualitative study

**DOI:** 10.1186/s12889-024-19182-6

**Published:** 2024-07-02

**Authors:** Yuanyuan Liu, Siyue Wei, Zhaoyu Cheng, Yidan Xian, Xuan Liu, Jie Yang, Yan Liu, Maohe Yu, Zhongdan Chen, Jiageng Chen, Jun Ma, Zhuang Cui, Changping Li

**Affiliations:** 1 Department of Epidemiology and Biostatistics, Tianjin Medical University, Tianjin, China; 2 Shenlan” Public Health Counseling Service Center, Tianjin, China; 3 STD & AIDS Control and Prevention Section, Tianjin Center for Disease Control and Prevention, Tianjin, China; 4 Tianjin Key Laboratory of Pathogenic Microbiology of Infectious Disease, Tianjin Centers for Disease Control and Prevention, Tianjin, 300011 China; 5 HIV/Hepatitis, STI/TB, World Health Organization Representative Office in China, 401 Dongwai Diplomatic Building 23, DongzhimenwaiDajie, Chaoyang District, Beijing, 100600 China; 6 Department of Epidemiology and Biostatistics, School of Public Health, Tianjin Medical University, Tianjin, China; 7 Tianjin Key Laboratory of Environment, Nutrition and Public Health, NO.22, Qixiangtai Street, Tianjin, 300041 China

**Keywords:** Pre-exposure prophylaxis, HIV, Cross-sectional study, MSM, Quantitative and qualitative study, PrEP cessation

## Abstract

**Background:**

Several studies have demonstrated the population-level effectiveness of oral PrEP in reducing the risk of HIV infection. However, oral PrEP utilization among MSM in China remains below 1%. While existing literature has primarily focused on oral PrEP preference and willingness, there is limited exploration of the underlying factors contributing to oral PrEP cessation in China. This study aims to fill this gap by investigating the factors associated with oral PrEP cessation among MSM in China.

**Methods:**

Assisted by MSM community organizations, we collected 6,535 electronic questionnaires from 31 regions across China, excluding Taiwan, Hong Kong, and Macau. The questionnaire focused on investigating MSM's awareness, willingness, usage, and cessation of oral PrEP. Additionally, 40 participants were randomly chosen for key informant interviews. These qualitative interviews aimed to explore the reasons influencing MSM discontinuing oral PrEP.

**Results:**

We eventually enrolled 6535 participants. Among the 685 participants who had used oral PrEP, 19.70% (135/685) ceased oral PrEP. The results indicated that individuals spending > ¥1000 on a bottle of PrEP (*aOR* = 2.999, *95% CI:* 1.886–4.771) were more likely to cease oral PrEP compared to those spending ≤ ¥1000. Conversely, individuals opting for on-demand PrEP (*aOR* = 0.307, *95% CI:* 0.194–0.485) and those using both daily and on-demand PrEP (*aOR* = 0.114, *95% CI:* 0.058–0.226) were less likely to cease PrEP compared to those using daily PrEP. The qualitative analysis uncovered eight themes influencing oral PrEP cessation: (i) High cost and low adherence; (ii) Sexual inactivity; (iii) Lack of knowledge about PrEP; (iv) Trust in current prevention strategies; (v) Poor quality of medical service and counseling; (vi) PrEP stigma; (vii) Partner and relationship factors; (viii) Access challenges.

**Conclusions:**

The cessation of oral PrEP among MSM in China is associated with various factors, including the cost of oral PrEP medication, regimens, individual perception of HIV risk, stigma, and the quality of medical services. It is recommended to provide appropriate regimens for eligible MSM and develop tailored combinations of strategies to enhance PrEP awareness and acceptance among individuals, medical staff, and the MSM community. The findings from this study can support the refinement of HIV interventions among MSM in China, contributing to efforts to reduce the burden of HIV in this population.

**Supplementary Information:**

The online version contains supplementary material available at 10.1186/s12889-024-19182-6.

## Introduction

Oral pre-exposure prophylaxis (PrEP) is a preventive approach involving daily or on-demand use of antiretroviral therapy to mitigate the risk of HIV transmission [1]. Both randomized clinical trials and real-world studies have evidenced the substantial efficacy of both daily and on-demand oral PrEP in diminishing the risk of HIV acquisition among high-risk cohorts, including men who have sex with men (MSM).

 [2–5]. A real-world study conducted in France and Canada observed an 86.0% reduction in HIV infection risk among MSM using on-demand PrEP compared to the placebo group [6]. Similarly, a randomized double-blind trial in Kenya and Uganda reported a relative reduction of 67% in HIV-1 incidence with once-daily tenofovir (95% *CI*, 44 to 81; *P* < 0.001) and a 75% reduction with combination tenofovir–emtricitabine (95% *CI*, 55 to 87; *P* < 0.001) [7]. In light of these findings, the World Health Organization strongly advocates the provision of oral PrEP to populations facing substantial risks of HIV transmission [8].

Available data suggest that the risk of HIV acquisition among gay men and other men who have sex with men was 22 times higher in 2018 than it was among all adult men [9]. In China, MSM accounted for 23.3% of newly reported HIV/AIDS cases in 2018 [10]. This situation is undoubtedly serious. Emtricitabine tenofovir tablets were approved by the Chinese State Drug Administration on August 11, 2020 [11]. China released its first expert consensus on pre-exposure prophylaxis (PrEP) for HIV infection by the end of the same year, serving as clinical technical guidance for the implementation of PrEP in our country. Oral PrEP was approved for marketing in foreign countries much earlier than in China, and the promotion and application of the drug have achieved remarkable results. In New South Wales, Australia, a large-scale roll-out of PrEP started on March 1, 2016, through a state-wide implementation research study [12]. HIV diagnoses in MSM in New South Wales declined from 295 in the 12 months before PrEP roll-out to 221 in the 12 months after (relative risk reduction: 25·1%, 95% CI: 10·5–37·4) [13]. Since the proven efficacy of Tenofovir Disoproxil Fumarate/Emtricitabine (TDF/FTC) for PrEP in 2010 [14] and its approval for use in the USA in 2012, PrEP use increased more than tenfold in the USA through 2017 [15]. Yet, of the estimated 8,226,000 MSM in China, less than 1% are using PrEP for HIV prevention [16]. Given this trend, promoting PrEP and understanding the barriers to PrEP use among MSM in China becomes an urgent need.

It is worth mentioning that most of the research on PrEP in China has been limited to regional willingness and preference surveys [17, 18]. To our knowledge, nationwide surveys of PrEP use have not been reported. Additionally, it's crucial to note that the adherence of subjects to medication significantly impacts the effectiveness of PrEP in preventing HIV infection [19]. There is a subset of the MSM population that discontinues PrEP due to a variety of barriers. Cessation of PrEP may be appropriate for some people who are no longer at risk of HIV infection; However, this is not the case for all patients who discontinue PrEP [20]. Some studies from Australia [21], Germany [22], and America [20] have explored the reasons affecting PrEP discontinuation, finding that high drug costs, concerns about potential side effects, and perceptions of low risk of HIV infection were common reasons for discontinuing PrEP [23]. Reasons for ceasing PrEP among China’s MSM are likely to differ from those in other countries, and to the best of our knowledge, they have not yet been reported.

This study examined the factors correlated with oral PrEP cessation among MSM in China. On this basis, the impact of oral PrEP regimens and medicine types on cessation was studied in depth from both quantitative and qualitative perspectives. Understanding the factors associated with and causes of oral PrEP cessation in MSM will help inform future oral PrEP implementation efforts.

## Methods

### Study design and participants

This cross-sectional study, conducted between October 20 and December 20, 2021, examined oral PrEP awareness, willingness, and use among MSM in 31 Chinese regions, comprising 22 provinces, 4 municipalities, and 5 autonomous regions. Sponsored by the WHO China Office, it was executed by the Tianjin Shenlan Public Health Counselling Service Center, with the assistance of MSM community organizations in distributing electronic questionnaires across geographical areas. For participant recruitment, a combination of online and offline methods was employed using convenience and snowball sampling. The online approach involved community workers publicizing and recruiting in WeChat Moments and Groups. The offline approach consisted of staff members visiting bars and bathhouses frequented by the gay community to recruit participants.

At the end of the questionnaire, participants were asked if they would like to participate in subsequent qualitative interviews. Those interested volunteered their contact information. Semi-structured interviews were conducted, and outlines were developed in advance by members of the qualitative analysis team. The interview outline drew upon salient theories and frameworks for understanding health behavior change, such as Social Cognitive Theory, the Theory of Planned Behavior [24], and the AIDS Risk Reduction Model [25]). The interview outline underwent continuous refinement through a literature review on PrEP awareness, willingness, acceptance, use, adherence, and persistence [18, 26, 27]. The semi-structured guides for Key Informant Interviews (KIIs) were designed to elicit discussion in these domains but also permit participants to discuss other, unanticipated topics relevant to the overall purposes of the research.

**Fig. 1:**
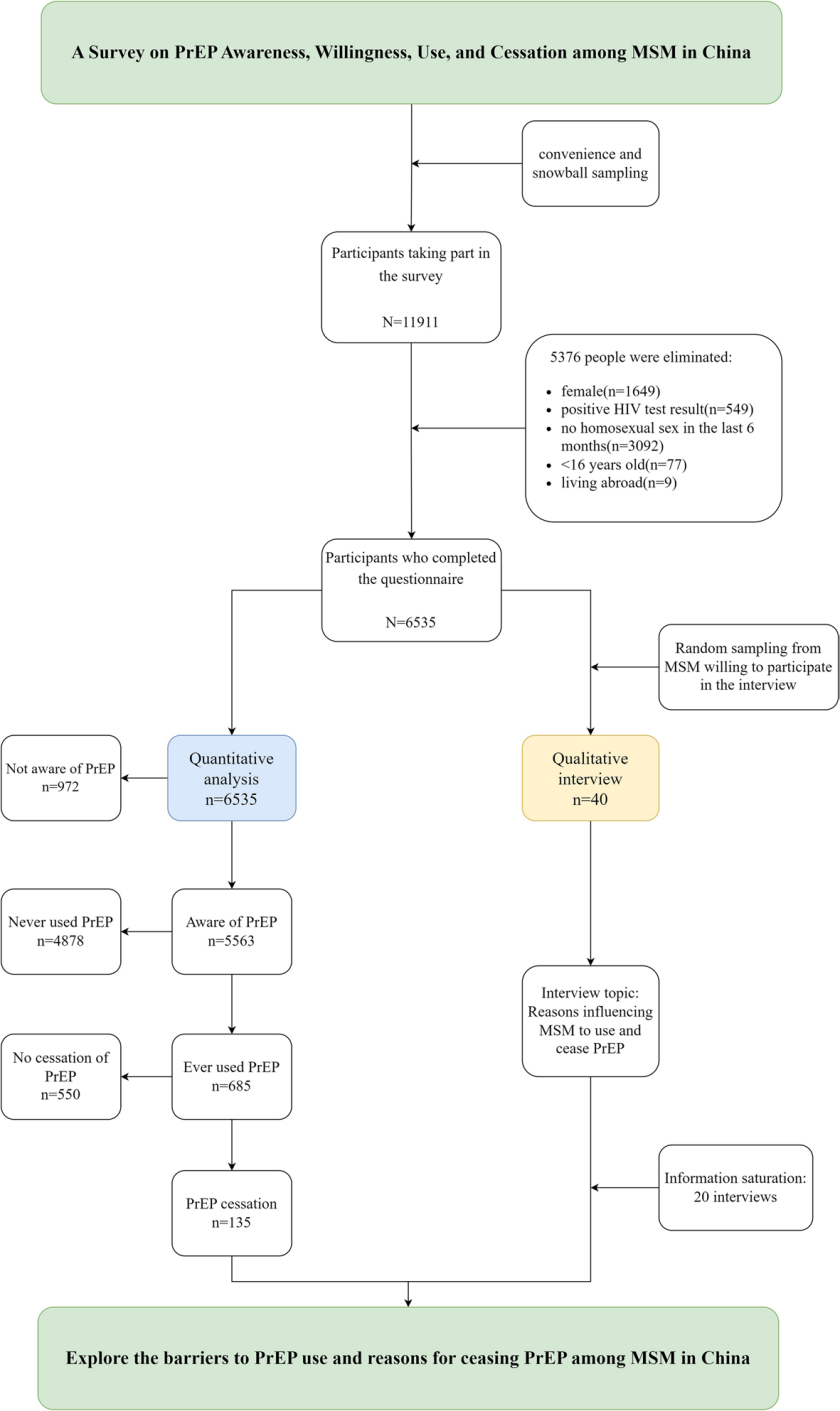
Study flowchart

Participants were eligible if they identified as males, were at least 16 years old, self-reported as HIV non-positive, resided in mainland China, and reported engaging in sexual activity with a man during the last six months. Detailed exclusion and inclusion criteria for participants can be found in Fig. 1. No remuneration was provided to participants for their participation in this survey.

### Study measures

Participants completed an electronic questionnaire regarding demographic characteristics, same-sex behavior, and PrEP-related information online using their personal devices. The questionnaire took, on average, 10 min to complete. Our primary outcome of interest was whether to cease oral PrEP, which was determined through two questions in the electronic questionnaire. Participants were asked the following: 'Why did you cease daily oral PrEP?' and 'Why did you cease on-demand oral PrEP?'. Participants were categorized as having ceased oral PrEP based on their responses to questions corresponding to their specific oral PrEP regimen.

Demographic characteristics included age, city of residence, educational attainment, and monthly income. Age categories were determined by referencing the age classification published by the WHO and previous literature [28–30]. Considering the participants' age range and the percentage distribution in each group, age was categorized as 16–24 years, 25–44 years, and 45 years and older. Educational attainment was dichotomized as junior high school and below, senior high school/ technical secondary school, college/ bachelor, and postgraduate and above. Based on previous studies, southwestern, northeastern, and northern China were considered high HIV prevalence areas [31]. Income monthly was categorized as having no regular income source, ≤ ¥3000, ¥3000–5000, ¥5000–8000, and ≥ ¥8000. Gross Domestic Product (GDP) serves as a measure of the economic situation and level of development of a region. According to the National Bureau of Statistics 2021 annual report, provinces, and cities ranking in the top ten in terms of per capita GDP are classified as high GDP regions, those ranking in the bottom ten are classified as low GDP regions, and the remaining areas are classified as moderate GDP regions [32].

Information on same-sex behavior included whether participants had engaged in providing sexual services to another person for money or property (commercial sex), whether they had been diagnosed with a sexually transmitted disease in the last year, their role in sexual activities in the last six months, condom usage during their most recent anal sex, the frequency of condom use during anal sex in the last six months, the number of sexual partners in the last six months, engagement in group sexual activity during the last six months, and awareness of the HIV status of their sexual partner in the last six months.

PrEP-related information encompassed whether participants were aware of oral PrEP, their willingness to use it, whether they had used oral PrEP in the past year, the cost of purchasing each bottle, instructions on how to take oral PrEP, and reasons for discontinuing its usage. The oral PrEP regimen, specifying how to take it, was classified as daily oral PrEP, on-demand oral PrEP, and both two regimens. From the question regarding the cost of purchasing a bottle of oral PrEP, a new variable—'the medication cost'—was derived. The medication cost was categorized as ≤ ¥1000 and > ¥1000.

### Sample size and sampling procedure

The sample size for this study was calculated using proportional sampling by taking the proportion of PrEP use (15%, derived from a pre-survey on PrEP use in Tianjin) and with the following assumptions: 2.0% margin of error and 95% level of confidence. Proportional sampling was conducted based on the number of males in each province, as recorded in the 2021 China Statistical Yearbook [33]. Considering a disqualification rate of 20% in survey responses, the sample size was adjusted to 5878. This adjusted sample size represented the minimum requirement for a random sampling approach. Taking into account online surveys, self-reporting, the need for additional subgroup analysis, and qualitative interviews, the sample size was further expanded by 10%. The equation represents the formula for calculating the sample size,

$$n=\frac{{\mu }_{\alpha /2}^{2}\pi (1-\pi )}{{\delta }^{2}}$$

From MSM who voluntarily participated in interviews, four categories were identified based on usage characteristics: those who wanted but did not take PrEP, those who did not want to take PrEP, those who ceased after using daily or on-demand PrEP, and those who ceased after using both daily and on-demand PrEP. Ten individuals were randomly selected from each category, totaling 40 participants, for Key Informant Interviews (KII) to gather crucial insights. According to the literature on qualitative research, data saturation was considered achievable after interviewing 20 MSM, as determined through group discussions [34].

### Ethical approval

This study received approval from the Institutional Review Board of Tianjin Medical University (approval number: TMUhMEC2021010). Participants were informed that their involvement was voluntary and anonymous, and they read the consent form online. Those who willingly agreed to take part in the survey proceeded by clicking a button to enter the online questionnaire.

### Statistical analysis

#### Quantitative analysis

We conducted a thorough verification process for outliers and missing values, and our data were found to be free of both. To enhance the analysis, we discretized the continuous variable (age) and recoded certain categorical variables, resulting in the creation of new variables (GDP, areas with high HIV prevalence, oral PrEP medicine type). Chi-square tests were employed to determine the significance of associations between the outcome variable and covariates (with a *p*-value < 0.05). Significant variables were included in multivariate logistic regression models, and results were expressed using adjusted odds ratios (*aOR*) and 95% confidence intervals (*CIs*). Stratified analyses were conducted by PrEP regimens to explore factors associated with PrEP cessation among MSM using different PrEP regimens. All statistical analyses were performed using SAS version 9.4 (SAS Institute Inc).

#### Qualitative analysis

The qualitative analysis team consisted of four members. Interviews were conducted one-on-one via WeChat voice calls. Participants were informed that the interviews were anonymous and agreed to have their voices recorded throughout the sessions. All participants were from mainland China, and the interviews were conducted in Chinese. The duration of each interview was limited to 30–45 min. Qualitative interview data was analyzed using thematic analysis [35]. The interview team developed the initial coding manual based on the interview outlines. After each interview, team members transcribed the audio recordings of the key informant interviews into text, incorporating the transcripts of the interview process, which were proofread during transcription. Throughout the transcription process, the coding manual was continuously revised for subsequent thematic analyses. The transcribed text was independently coded by two members using Atlas. ti 8.3 and a third analyst then reviewed inter-coder consistency. The coded text and underlying themes were discussed and agreed upon by the research team, ultimately presenting the frequency and co-occurrence of themes. After completing the qualitative analyses, the interviews were collectively translated by team members with a high level of English proficiency. Additional members, specializing in English, were invited to verify the translations. Finally, a team meeting was conducted to finalize and review the translations.

## Result

### Quantitative findings

**Fig. 2:**
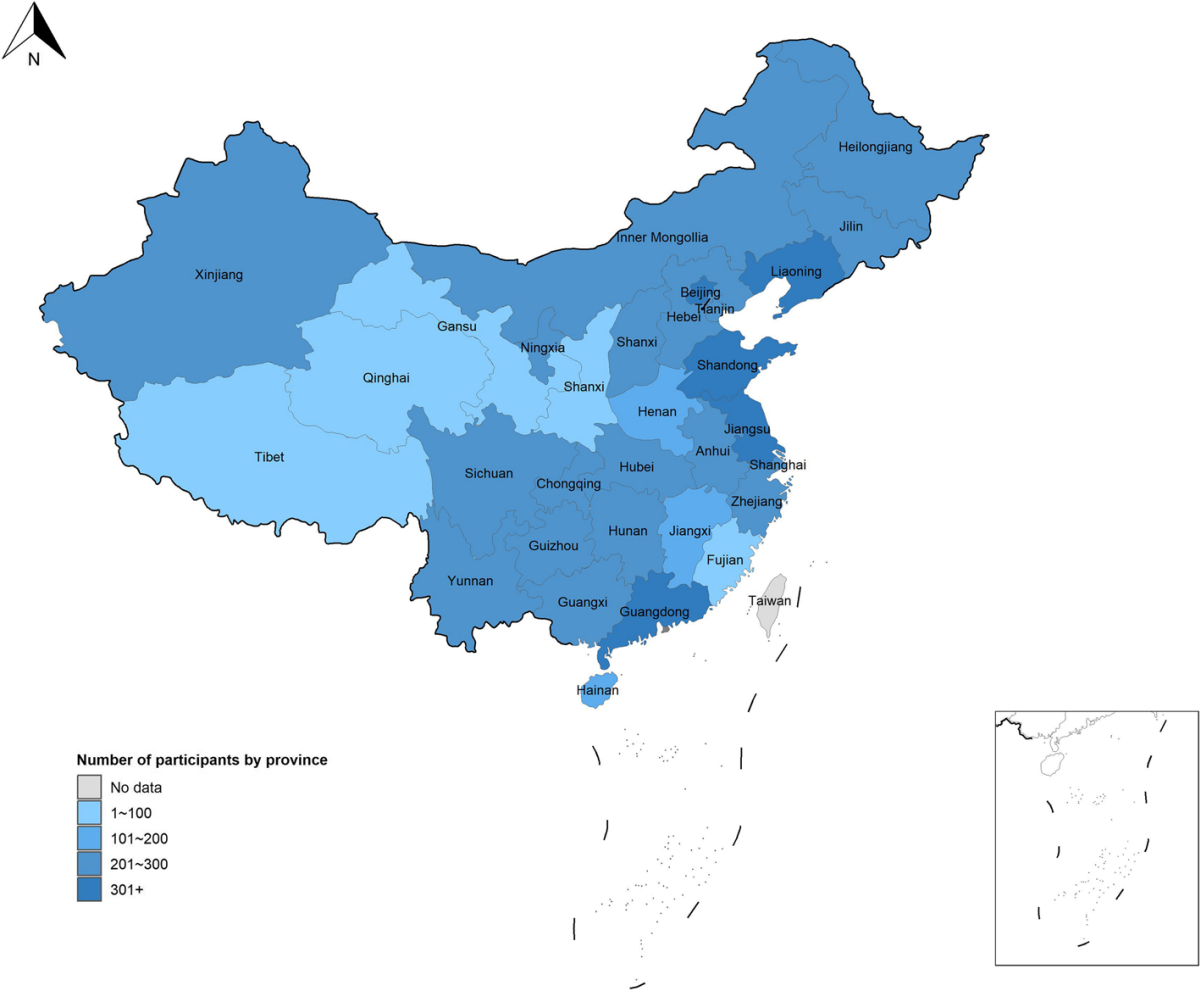
The distribution of people participating in this survey across regions in China

This national survey ultimately included 6,535 participants. Figure 2 illustrates the distribution of people participating in this PrEP survey by province in China. Among the 5,563 participants who were aware of oral PrEP, 12.3% (685/5,563) had used it, and within this group, 19.7% (135/685) had discontinued oral PrEP.

### Sample characteristics

Overall, of all MSM who ever used PrEP, 71.5% were aged 25–44 years, and 83.7% had a college/bachelor's degree and above. More than half earned ¥5000 or more a month, nearly half lived in areas with a high prevalence of HIV, and nearly 80% lived in high GDP areas (Table 1).

**Table 1:** Characteristics of MSM participants using oral PrEP in China

**Characteristic**	**Total** (*N* = 685)	**Cessation** (*N* = 135)	**No cessation** (*N* = 550)	*χ*^2^	*P* value
Economic Level Division				0.6865	0.7095
High GDP	256 (37.4)	48 (35.6)	208 (37.8)		
Medium GDP	288 (42.0)	61 (45.2)	227 (41.3)		
Low GDP	141 (20.6)	26 (19.2)	115 (20.9)		
Areas with high HIV prevalence				0.7671	0.3811
No	358 (52.3)	66 (48.9)	292 (53.1)		
Yes	327 (47.7)	69 (51.1)	258 (46.9)		
Age(years)				0.6723	0.7145
16–24	157 (22.9)	29 (21.5)	128 (23.3)		
25–44	490 (71.5)	100 (74.1)	390 (70.9)		
45 and older	38 (5.6)	6 (4.4)	32 (5.8)		
Educational attainment				5.2402	0.1550
Junior High School and below	35 (5.1)	9 (6.7)	26 (4.7)		
Senior High School/ Technical Secondary School	77 (11.2)	13 (9.6)	64 (11.6)		
College/ Bachelor	458 (66.9)	98 (72.6)	360 (65.5)		
Postgraduate and above	115 (16.8)	15 (11.1)	100 (18.2)		
Monthly income				2.0546	0.7257
No regular income source	104 (15.2)	23 (17.1)	81 (14.7)		
≤ ¥3000	53 (7.7)	9 (6.7)	44 (8.0)		
¥3000-¥5000	152 (22.2)	30 (22.2)	122 (22.2)		
¥5000-¥8000	147 (21.5)	33 (24.4)	114 (20.7)		
≥ ¥8000	229 (33.4)	40 (29.6)	189 (34.4)		
Commercial sexual behavior				1.0398	0.3079
No	607 (88.6)	123 (91.1)	484 (88.0)		
Yes	78 (11.4)	12 (8.9)	66 (12.0)		
Anal sex roles within the previous 6 months				7.3660	0.0611
Top	231 (33.7)	49 (36.3)	182 (33.1)		
Versatile	187 (27.3)	46 (34.1)	141 (25.6)		
Bottom	242 (35.3)	35 (25.9)	207 (37.7)		
No anal sex	25 (3.7)	5 (3.7)	20 (3.6)		
Condom use during last anal sex(N = 660)				14.1863	0.0002
No	190 (28.8)	20 (15.4)	170 (32.1)		
Yes	470 (71.2)	110 (84.6)	360 (67.9)		
Number of sex partners within the previous 6 months				0.1869	0.9108
1–5	517 (75.5)	101 (74.8)	416 (75.7)		
6–10	89 (13.0)	19 (14.1)	70 (12.7)		
≥ 11	79 (11.5)	15 (11.1)	64 (11.6)		
Group sexual activity within the previous 6 months				0.9095	0.3402
No	495 (72.3)	102 (75.6)	393 (71.5)		
Yes	190 (27.7)	33 (24.4)	157 (28.5)		
Knowledge of the HIV status of current sex partners				0.8678	0.6480
Know all	272 (39.7)	51 (37.8)	221 (40.2)		
Partially know	299 (43.7)	58 (43.0)	241 (43.8)		
Don’t know	114 (16.6)	26(19.2)	88 (16.0)		
Latest HIV test result				2.8164	0.0933
Don’t know	20 (2.9)	1 (0.74	19 (3.5)		
HIV negative	665 (97.1)	134 (99.3)	531 (96.5)		
Suffering from STDs in the last year				0.2262	0.6343
Yes	88 (12.9)	19 (14.1)	69 (12.6)		
No	597 (87.1)	116 (85.9)	481 (87.4)		
Oral PrEP medication cost				26.0036	< 0.0001
≤ ¥1000	545 (79.6)	86 (63.8)	459 (83.4)		
> ¥1000	140 (20.4)	49 (36.2)	91 (16.6)		
Oral PrEP regimen				58.4190	< 0.0001
Daily oral PrEP	143 (20.9)	59 (43.7)	84 (15.3)		
On-demand oral PrEP	371 (54.1)	62 (46.0)	309 (56.5)		
Both Regimens	171 (25.0)	14 (10.3)	157 (28.5)		

The contents of the parentheses indicate column percentages

In univariate analyses, compared to those who did not cease PrEP, the majority of participants who ceased PrEP used condoms during their last anal sex (84.6% vs. 67.9%, *p* = 0.0002), spent > ¥1000 on a bottle of PrEP (36.2% vs. 16.6%, *p* < 0.0001), and had only ever used daily PrEP (43.7% vs. 15.3%, *p* < 0.0001) (Table 1).

### Factors correlated with the cessation of oral PrEP

In multivariate analysis, three models were constructed using logistic regression analysis. Model 1 incorporated only the oral PrEP regimen and oral PrEP medicine type, Model 2 incorporated demographic characteristics based on Model 1, and Model 3 incorporated same-sex behavior characteristics based on Model 2. Model 3 results showed that those who spent > ¥1000 on a bottle of PrEP(*aOR* = 2.999, *95%CI:* 1.886–4.771) were likely to cease oral PrEP, compared to those who spent ≤ ¥1000 on a bottle of PrEP; compared to those who ever used daily oral PrEP, those who ever used on-demand oral PrEP (*aOR* = 0.307, *95%CI:* 0.194–0.485) and those who ever used both oral PrEP regimens (*aOR* = 0.114, *95%CI:* 0.058–0.226) were less likely to cease oral PrEP (Table 2).

**Table 2:** Adjusted correlations with PrEP cessation among MSM participants using PrEP

Variable	Model 1		Model 2		Model 3	
	***aOR(95%CI)***	*P*** value**	*aOR(95%CI)*	*P*** value**	*aOR(95%CI)*	*P*** value**
PrEP medication cost						
≤ ¥1000	reference		reference		reference	
> ¥1000	2.847 (1.827,4.436)	< 0.0001	3.040 (1.935,4.774)	< 0.0001	2.999 (1.886,4.771)	< 0.0001
PrEP regimen						
Daily oral PrEP	reference		reference		reference	
On-demand oral PrEP	0.310 (0.199,0.481)	< 0.0001	0.317 (0.203,0.495)	< 0.0001	0.307 (0.194,0.485)	< 0.0001
Both regimens	0.124 (0.065,0.238)	< 0.0001	0.116 (0.060,0.226)	< 0.0001	0.114 (0.058,0.226)	< 0.0001

Model 1 incorporated only PrEP-related variables as covariates: PrEP medication cost and PrEP regimen. Model 2 built upon Model 1 by including demographic characteristics as covariates: age, economic level division, monthly income, and whether the areas had high HIV prevalence. Model 3 extended Model 2 by including same-sex behavior variables as covariates: the number of people who have had sexual activity, whether or not they have engaged in group sexual activity within the previous 6 months, and whether or not they have suffered from other STDs in the last year

Table 2 shows statistical information for only two variables, PrEP medication cost and PrEP regimen, and details of the other variables for the three models constructed are shown in Table S1

Table S1 results showed that individuals who used condoms during their last anal sex (*aOR* = 2.450, *95%CI:* 1.425–4.210) were more likely to cease oral PrEP, compared to those who didn’t use condoms. Additionally, individuals who knew that their HIV test result was negative (*aOR* = 8.178, *95%CI:* 1.026–65.209) were more likely to cease oral PrEP, compared to those who didn’t know their HIV test result (Table S1).

### Results of stratified analysis

**Fig. 3:**
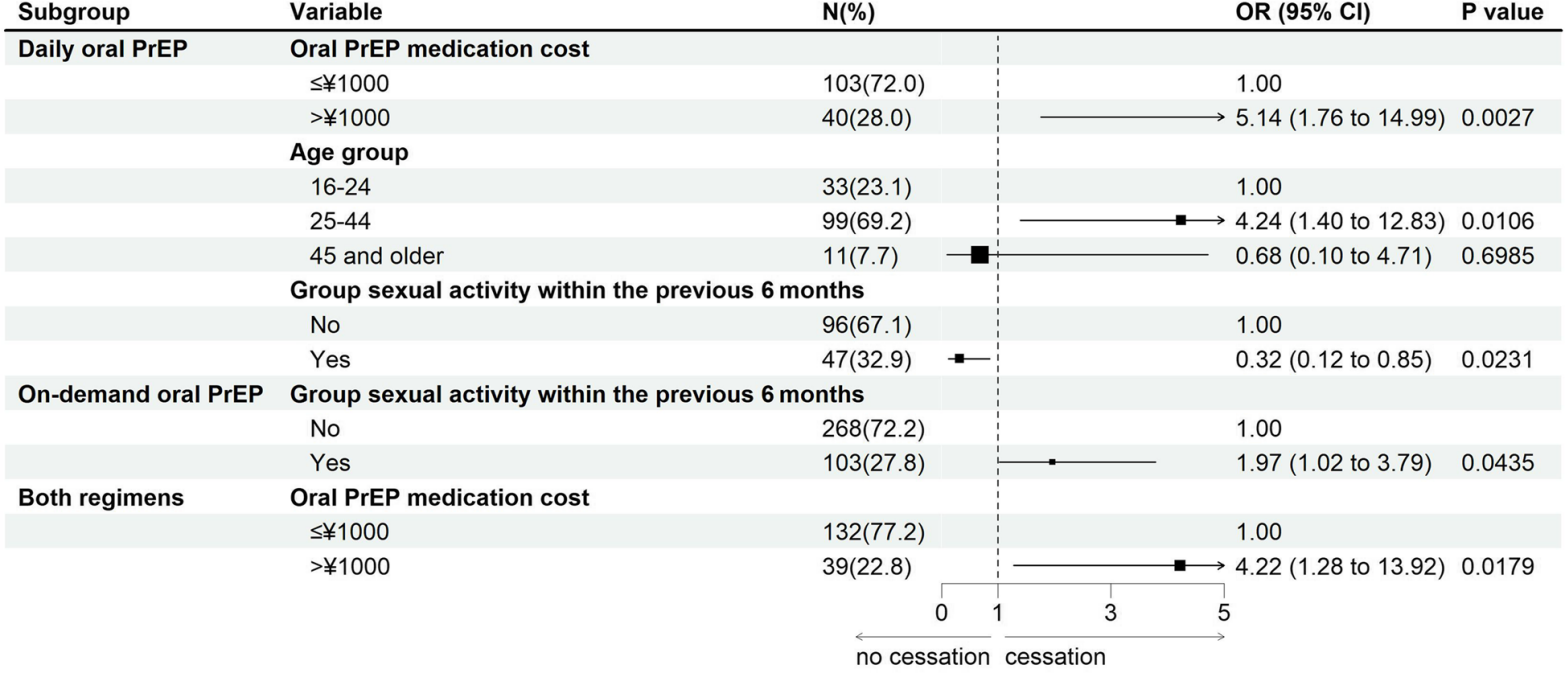
Results from Stratified Analysis by PrEP Regimen

Subgroup analysis was performed stratified by oral PrEP regimen. Among those who had used daily oral PrEP, the results showed that those who spent > ¥1000 on a bottle of PrEP (*aOR* = 5.14, *95%CI:* 1.76, 14.99), and those in the 25–44 age group (*aOR* = 4.24, *95%CI:* 1.40, 12.83) were more likely to cease oral PrEP; those who had group sexual activity in the last 6 months (*aOR* = 0.32, *95%CI:* 0.12, 0.85) were less likely to cease oral PrEP. Among those who had used on-demand oral PrEP, those who had group sexual activity in the last 6 months (*aOR* = 1.97, *95%CI:* 1.02, 3.79) were more likely to cease PrEP. Among those who had used both oral PrEP regimens, those who spent > ¥1000 on a bottle of PrEP (*aOR* = 4.22, *95%CI:* 1.28, 13.92) were more likely to cease oral PrEP (Fig. 3).

### Qualitative findings

We interviewed 20 MSM. Participants ranged in age from 16 to 44 years old, 60.0% were aged 25–44 years, and 85.0% had a college/bachelor's degree. 75.0% earned ¥5000 or more a month, 60.0% lived in areas with a high prevalence of HIV, and 40.0% lived in high-GDP cities. 45% wanted but did not take oral PrEP, 20% did not want to take oral PrEP, 15% ceased PrEP after using daily or on-demand oral PrEP and 20% ceased oral PrEP after both using daily and on-demand oral PrEP (Table 3).

**Table 3:** Characteristics of the sample participating in qualitative interviews

Characteristic	Number (*N* = 20)	(%)
Economic Level Division		
High GDP	8	40
Medium GDP	7	35
Low GDP	5	25
Areas with high HIV prevalence		
No	8	40
Yes	12	60
Age(years)		
16–24	8	40
25–44	12	60
Educational attainment		
Senior High School/ Technical Secondary School	3	15
College/ Bachelor	17	85
Monthly income		
No regular income source	4	20
≤ ¥5000	1	5
¥5000-¥8000	8	40
≥ ¥8000	7	35
Commercial sexual behavior		
No	19	95
Yes	1	5
Suffering from STDs in the last year		
No	12	60
Yes	8	40
Membership		
People who wanted but did not take oral PrEP	9	45
People who did not want to take oral PrEP	4	20
People who ceased after using daily or on-demand oral PrEP	3	15
People who ceased after using both daily and on-demand oral PrEP	4	20

MSM described what they felt were the most important elements influencing whether they used, continued to use, or ceased PrEP. The qualitative analysis of this survey found eight main barriers to PrEP use: (i) High cost and low adherence; (ii) Sexual inactivity; (iii) Lack of knowledge about PrEP; (iv) Trust in current prevention strategies; (v) Poor quality of medical service and counseling; (vi) PrEP stigma; (vii) Partner and relationship factors; (viii) Access challenges. Table 4 shows a detailed explanation of the eight themes.

**Table 4:** Results of the qualitative analysis: eight themes

Theme	Interpretation
High cost and low adherence	The price of PrEP is unaffordable Difficulty in adhering to PrEP
Sexual inactivity	Decreased frequency of sexual behavior
Lack of knowledge about PrEP	Lack of understanding about conditions for taking medication Lack of understanding about the preventive effects and side effects of PrEP
Trust in current preventive strategies	Stick to condoms Having a stable sexual relationship
Poor quality of medical service and counselling	Poor professional guidance from doctors Indifference and impatience of those providing PrEP counselling services
PrEP stigma	Fear of receiving discrimination and rejection
Partner and relationship factors	Desire to gain the trust of sexual partners Fear of sexual partners mistaking them for HIV-infected people
Access challenges	Difficulty in accessing oral PrEP and related counselling services

### High cost and low adherence

Our interviews indicated that the price of oral PrEP and the challenge of adhering to the medication were significant reasons for MSM to discontinue oral PrEP. Participants cited factors such as the unaffordable cost of oral PrEP, the need for advance preparation for on-demand oral PrEP, and the difficulty of maintaining adherence to daily oral PrEP. Based on these considerations, they made the decision to discontinue oral PrEP.

One participant who wanted but did not take PrEP shared his perspective: *“I can't guarantee that I'll be able to take my medication on time, as the on-demand PrEP regimen requires preparation. If the daily PrEP involves taking it every day, I might not be able to stick with it.”*

Another participant, who ceased after using on-demand oral PrEP, attributed it to the unaffordable price of oral PrEP: “*In terms of the original research drugs, it's about 1980 yuan per month if taken daily, which I find a bit burdensome. Although the cost decreases with domestic drugs, around 350 yuan for a bottle, there might be concerns about their quality and preventive effectiveness.”*

### Sexual inactivity

Participants perceived themselves to be less sexually active and therefore would use oral PrEP less often. A man who ceased after using on-demand oral PrEP shared his opinion: *"Recently, I haven't been in a relationship and haven't had many friends around, which has led to a decrease in my sexual activity. Consequently, I've adjusted the frequency of my PrEP usage to match the reduced frequency of my sexual behavior."*

### Lack of knowledge about PrEP drugs

Participants also noted that factors influencing their use of PrEP were related to a lack of understanding of oral PrEP. MSM often found themselves unaware of the conditions under which oral PrEP is taken, how it is taken, and its preventative and side effects, and were therefore hesitant to take it freely. As mentioned by a participant who wanted but did not take PrEP: “*Since I've never actually seen the medication in person and am not familiar with the instructions for its use, I'm hesitant to try it out."*

Additionally, concerns about the side effects of oral PrEP are an important factor. MSM believed that the drug had numerous side effects and that long-term use was not good for health. One participant who did not want to use PrEP mentioned: *“I do have some concerns about the medication itself because, regardless of the type, long-term use of any drug is not good for the body. Therefore, even if I choose to take it later, it won't be on a long-term basis.”*

One participant, who initially considered taking PrEP but refrained from doing so, highlighted the inadequacy of the drug's promotional efforts, leading to a lack of awareness among many individuals about where to purchase it. This participant suggested that enhancing the promotion and support of PrEP by medical and community institutions would provide reassurance to MSM, encouraging them to opt for PrEP.

### Trust current preventive strategies

Participants were more inclined to trust current prevention strategies, such as consistently using condoms, having a regular sexual partner, and knowing the HIV status of their sexual partner, compared to using PrEP. One participant stated that he would use a condom in every sexual encounter and would also inquire about the other person's HIV status before engaging in sex.

Another participant who wanted but did not take oral PrEP said: *“We are committed partners with a relatively stable relationship. Both of us have undergone health checks, and the results were negative. Moreover, our sexual lifestyle is consistent and we are both stable individuals. Therefore, we are not considering taking PrEP for now.”*

#### Poor quality of medical service and counseling

Our interviews revealed a factor that had not been considered previously: participants' perception of poor quality medical service and counseling. This was attributed to doctors' misconceptions and biases about PrEP, leading to their inability to provide proper guidance to MSM. The participant, who ceased after using both daily and on-demand PrEP, remarked, *“The doctor told me that even if I take the medicine, there's still a chance of infection. Moreover, the medication itself has some toxicity; it can harm the liver and kidneys. Why not take control of your own actions, and find a committed partner, where both parties are aware of each other's background? Why resort to taking that medicine? It's essentially useless and harms the body.”*

In addition, poor-quality PrEP-related counseling services were also a significant issue mentioned by participants. MSM found that when consulting with relevant personnel about PrEP-related issues, they encountered indifference and impatience, with personnel being unable to clearly address their queries. As mentioned by a participant who ceased after using on-demand PrEP: *“During the consultation, the doctor seemed a bit impatient, and he didn't address my concerns directly. I had listed all my questions, numbering them one by one, but he didn't respond to them in order. Instead, he provided a general overview, leaving me with doubts. Furthermore, his attitude wasn't very good, and he didn't inquire further.”*

#### PrEP stigma

Some participants mentioned PrEP stigma as a reason for not taking their medication, and MSM was concerned about being mistaken for HIV-infected people due to the “therapeutic effect” of the drug’s instructions. One participant, who wanted but did not take PrEP, shared his opinion: *“If the name or the instructions of the medication mentioned ‘prevention’, I might be more open to accepting this kind of medicine. Otherwise, if it's labeled as 'treatment', my family might see it and suspect that I have some health issues.”*

Additionally, since the medication is dispensed at designated HIV hospitals, MSM were concerned about being seen by acquaintances who might mistakenly assume they are HIV-positive. An interviewee who wanted but did not take PrEP shared, *“I feel that if I were to buy PrEP at an AIDS treatment center, there's a chance of encountering someone I know. I worry that they might think I'm an HIV-positive individual. Being seen in such a place could easily lead to misunderstandings.”*

#### Partner and relationship factors

Participants also mentioned choosing not to take oral PrEP because of their sexual partners. Participants felt that if they chose to take PrEP it was a sign of distrust of their sexual partner. They wanted to find genuine sexual partners and gain their trust.

As mentioned by a participant who wanted but did not take PrEP: *“For people like us, it's relatively difficult and quite rare to find someone who genuinely wants to engage in a heartfelt relationship. But now, I completely trust him, and he loves and trusts me deeply. This gives me a sense of security and trust. I don't harbor doubts. If there ever comes a day when I'm faced with a situation like contracting HIV, I believe I would accept it.”*

#### Access challenges

Participants identified difficulties in accessing oral PrEP as an important factor influencing them not to take their medication, including: not being able to prepare oral PrEP in advance, complicated procedures for picking up their medication at the hospital, inconvenience in purchasing the medication, and wasted time.

One participant who ceased after using both daily and on-demand PrEP said: *"I find it inconvenient to buy PrEP because the channels for purchasing it are quite limited right now. For instance, in the city where I live, my friend has to travel over 10 km to get the medication. It's a relatively long distance, and for the time being, it might be the only place where the drug is available."*

## Discussion

This study aimed to investigate the factors influencing oral PrEP cessation among MSM in China using a mixed-methods approach. The type and regimen of oral PrEP are important factors influencing MSM to cease PrEP, as revealed by quantitative analysis. In our study, 19.7% (135/685) had ceased oral PrEP. However, an Australian study showed that 14% (140/970) had stopped using oral PrEP [21]. PrEP cessation in China was higher than abroad, necessitating further exploration of factors and reasons related to oral PrEP cessation.

This study demonstrated that individuals who had used daily oral PrEP were more likely to cease PrEP. This tendency may be associated with the challenges of adhering to a daily regimen and the associated costs [36]. Taking on-demand oral PrEP as an alternative to the daily regimen reduces pill burden, drug exposure, and overall cost. Qualitative analysis found that MSM discontinued PrEP due to concerns about medication side effects. One study found that people who use on-demand PrEP have less concern about the risk of side effects [22]. Therefore, adopting on-demand PrEP may represent an effective strategy to alleviate users' concerns about side effects compared to the daily regimen. Qualitative interviews found MSM not using or ceasing PrEP due to various factors, including sexual inactivity, high cost, and low adherence. This aligns with the findings of two US studies where most oral PrEP users switched from daily PrEP to on-demand PrEP due to decreased frequency of sexual activity, a preference for fewer pills, a need to reduce costs, and a desire to minimize side effects [37, 38]. Other forms of drug delivery, such as injectable PrEP or implants, are available in other countries and are currently under development for use in China [39]. These alternative methods may be more suitable for this group with poor adherence in the future. We recommend that health authorities issue guidelines on oral PrEP promptly for doctors, community organizations, and MSM, providing detailed information on the two oral PrEP regimens to help MSM make informed choices based on their real-life situations.

This study revealed that individuals spending > ¥1000 on a bottle of PrEP were more likely to cease PrEP compared to those spending ≤ ¥1000. This trend was particularly noticeable among those taking daily oral PrEP, perhaps due to the increased pill count and cost. High medication costs and related services were cited as reasons for ceasing PrEP, supported by qualitative interviews where participants mentioned cost burdens. These findings align with cross-sectional studies determining that PrEP price is a main barrier to its uptake [40–42]. A study in the Netherlands found that although the introduction of generic formulations of PrEP led to a decrease in the price of PrEP, the less well-off group continued to find the price of €50 per month unaffordable [43]. A US study found that groups with insurance were four times more likely to use PrEP than those without insurance [44]. The high price of oral PrEP remains a significant reason for MSM to cease its use, especially for those in poorer financial situations. To address this issue, we recommend considering the affordability for the target population when setting PrEP prices and including PrEP in health insurance reimbursement coverage.

In our study, participants who used condoms during their last sexual behavior were more likely to cease oral PrEP. The stratified analysis revealed that among daily oral PrEP users, those who had not engaged in group sexual activity in the past six months were more likely to cease PrEP. Additionally, qualitative studies with interviewers suggested stopping oral PrEP due to a belief in current prevention strategies. This may be associated with a low self-perception of risk, defined as the risk of acquiring HIV in the past six months [45]. Low self-perceived risk has been recognized as a barrier to successful PrEP implementation [46]. Biello’s study found that a high self-perceived risk was significantly associated with multiple sex partners [47]. A Toronto study discovered that reduced condom use corresponded to an increased perceived HIV risk [48]. However, there is a disconnect between self-perceived risk and actual risk [49], and changes in self-perceived risk may lead to PrEP cessation regardless of actual changes in risk behavior. It is imperative to clarify that we focused on those with high actual HIV risk but low self-perceived risk. MSM may also choose to cease PrEP for other reasons (e.g., starting a monogamous relationship, etc.), but these reasons were not explored in this study. In addition, this study also found that among those using on-demand oral PrEP, those who had multi-person sexual behavior in the last 6 months were more likely to cease oral PrEP. This may be due to the more frequent and random occurrence of sexual behavior in this group, who are unable to manage to take oral PrEP before an unknown sexual behavior, and for whom the use of daily oral PrEP is a better option. Safely discontinuing PrEP also requires greater understanding around the duration of daily dosing needed for protection after last HIV exposure, and clear strategies to re-engage persons as their HIV exposure risk changes overtime. In summary, when MSM consider stopping oral PrEP, it is recommended they seek a risk assessment from a community organization or doctor rather than merely relying on self-assessment. Furthermore, there is a need to develop decision-support tools around cessation and restart of PrEP that capture the complex relationship between risk perceptions and risk behaviors.

The qualitative interviews also revealed implicit psychosocial factors contributing to MSM ceasing oral PrEP, complementing the findings of the quantitative study. Some interviewees expressed dissatisfaction with service providers, citing inadequate answers to their questions and a perceived poor attitude. Therefore, it is recommended that staff members undergo uniform training to enhance their ability to communicate patiently with MSM and address any confusion they may have before initiating oral PrEP. Qualitative interviews brought to light PrEP-related stigma and pressure to access PrEP drugs as reasons for cessation within the MSM group. PrEP-related stigma and shaming present potential barriers to PrEP implementation and maintenance [50]. Based on these findings, it is recommended to launch educational campaigns on oral PrEP for all populations to promote a positive attitude towards PrEP and reduce the associated stigma within the general public, which is crucial for the successful implementation of oral PrEP.

### Strengthens and limitations

We believe that the highlights of this study are as follows: (1) It represents the first nationwide survey in China on oral PrEP use, adherence, and cessation, featuring a large sample size (6535 participants) and representative data; (2) This study is the first to explore factors associated with oral PrEP cessation among MSM in China; (3) It integrates both quantitative and qualitative approaches to address the limitations of a singular study type, allowing the results from both methods to corroborate and complement each other.

This study has some limitations. First, its cross-sectional design prevents the determination of the temporal order of certain associations, necessitating longitudinal studies for conclusive evidence. Second, due to the highly stigmatized and hidden nature of the MSM population, it is likely that the study participants belong to the more open MSM (excluding closeted groups). This represents a potential limitation of our study, as the results are only transferable to populations in similar contexts. Additionally, self-reported data may introduce social desirability and recall biases. The web-based survey may exhibit volunteer bias, as those knowledgeable about oral PrEP were more likely to participate, potentially leading to an overestimation of findings. Finally, the number of MSM we interviewed does not permit population-level generalizations. However, through in-depth, semi-structured interviewing, we gained insights into issues affecting oral PrEP cessation.

In the future, we will observe and analyze the transition between the two PrEP regimens and explore the factors influencing this shift in MSM behavioral patterns. Additionally, we have established a follow-up cohort in Tianjin, with plans to expand this cohort to other provinces and cities across China. This expansion will enable us to track the dynamics of PrEP awareness, willingness, usage, and cessation.

## Conclusion

The cessation of oral PrEP among MSM in China is associated with various factors, including the cost of oral PrEP medication, regimens, individual perception of HIV risk, stigma, and the quality of medical services. It is recommended to provide appropriate regimens for eligible MSM and develop tailored combinations of strategies to enhance PrEP awareness and acceptance among individuals, medical staff, and the MSM community. The findings from this study can support the refinement of HIV interventions among MSM in China, contributing to efforts to reduce the burden of HIV in this population.

### Supplementary Information

Supplementary Material 1.

## Data Availability

The data that support the findings of this study are available from Tianjin “Shenlan” Public Health Counselling Service Centre but restrictions apply to the availability of these data, which were used under license for the current study, and so are not publicly available. Data are however available from the authors upon reasonable request and with permission of Tianjin “Shenlan” Public Health Counselling Service Centre. If anyone would like to request data from this study, please contact Cuizhuang@tmu.edu.cn.

## Abbreviations

PrEP: Pre-exposure prophylaxis
MSM: Men who have sex with men
CI: Confidential interval
TDF: Tenofovir Disoproxil Fumarate
FTC: Emtricitabine
