# Supplementary material for: Correlates of oral pre-exposure prophylaxis cessation among men who have sex with men in China: implications from a nationally quantitative and qualitative study
Source: BMC Public Health. 2024 Jul 2;24:1765. doi: 10.1186/s12889-024-19182-6 (PMC11221119; doi:10.1186/s12889-024-19182-6)
Supplement: Supplementary file 1 — Supplementary Material 1. [file 12889_2024_19182_MOESM1_ESM.docx]

**Table S1 Adjusted correlations with PrEP cessation among MSM participants using PrEP**

| **Model** | **Variable** | | **Ref** | **Estimate** | **Standard error** | **Wald** $\boldsymbol{x}\boldsymbol{2}$ | ***P* value** | ***OR(95%CI)*** |
| --- | --- | --- | --- | --- | --- | --- | --- | --- |
| **Model 1** | **Oral PrEP medicine type** | | vs.Domestic medicine |  |  |  |  |  |
|  |  | Imported medicine |  | 1.0461 | 0.2263 | 21.3659 | <0.0001 | 2.847(1.827,4.436) |
|  | **Oral PrEP regimen** | | vs.Daily oral PrEP |  |  |  |  |  |
|  |  | On-demand oral PrEP |  | -1.1723 | 0.2248 | 27.2014 | ＜0.0001 | 0.310(0.199,0.481) |
|  |  | Both regimens |  | -2.0876 | 0.3325 | 39.4111 | ＜0.0001 | 0.124(0.065,0.238) |
| **Model 2** | **Oral PrEP medicine type** | | vs.Domestic medicine |  |  |  |  |  |
|  |  | Imported medicine |  | 1.1117 | 0.2303 | 23.2992 | <0.0001 | 3.040(1.935,4.774) |
|  | **Oral PrEP regimen** | | vs.Daily oral PrEP |  |  |  |  |  |
|  |  | On-demand oral PrEP |  | -1.1483 | 0.2272 | 25.5437 | ＜0.0001 | 0.317(0.203,0.495) |
|  |  | Both regimens |  | -2.1055 | 0.3330 | 39.9818 | ＜0.0001 | 0.116(0.060,0.226) |
|  | **Educational attainment** | | vs.Junior High School and below |  |  |  |  |  |
|  |  | Senior High School/ Technical Secondary School |  | -0.9218 | 0.5363 | 2.9542 | 0.0857 | 0.398(0.139,1.138) |
|  |  | College/ Bachelor |  | -0.3997 | 0.4388 | 0.8296 | 0.3624 | 0.671(0.284,1.585) |
|  |  | Postgraduate and above |  | -1.0748 | 0.5139 | 4.3751 | 0.0365 | 0.341(0.125,0.935) |
| **Model 3** | **Oral PrEP medicine type** | | vs.Domestic medicine |  |  |  |  |  |
|  |  | Imported medicine |  | 1.0984 | 0.2368 | 21.5182 | <0.0001 | 2.999(1.886,4.771) |
|  | **Oral PrEP regimen** | | vs.Daily oral PrEP |  |  |  |  |  |
|  |  | On-demand oral PrEP |  | -1.1815 | 0.2340 | 25.5018 | ＜0.0001 | 0.307(0.194,0.485) |
|  |  | Both regimens |  | -2.1700 | 0.3484 | 38.8036 | ＜0.0001 | 0.114(0.058,0.226) |
|  | **Condom use during last anal sex** | | vs.NO |  |  |  |  |  |
|  |  | YES |  | 0.8960 | 0.2763 | 10.5150 | 0.0012 | 2.450(1.425,4.210) |
|  | **Latest HIV test result** | | vs.Don’t know |  |  |  |  |  |
|  |  | HIV negative |  | 2.1015 | 1.0593 | 3.9361 | 0.0473 | 8.178(1.026,65.209) |

Note: Model 1 incorporated only PrEP-related variables as covariates: PrEP medicine type and PrEP regimen. Model 2 built upon Model 1 by including demographic characteristics as covariates: age, economic level division, monthly income, and whether the areas had high HIV prevalence. Model 3 extended Model 2 by including homosexual behavior variables as covariates: the number of people who have had sexual activity, whether or not they have engaged in group sexual activity within the previous 6 months, and whether or not they have suffered from other STDs in the last year.
